# The Potential of Tumor Debulking to Support Molecular Targeted Therapies

**DOI:** 10.3389/fonc.2020.00801

**Published:** 2020-06-18

**Authors:** Felix Oppel, Martin Görner, Holger Sudhoff

**Affiliations:** ^1^Department of Otolaryngology, Head and Neck Surgery, Klinikum Bielefeld, Bielefeld, Germany; ^2^Department of Hematology and Oncology, Klinikum Bielefeld, Bielefeld, Germany

**Keywords:** precision oncology, molecular targeting, clonal heterogeneity, therapy resistance, cancer therapy, cancer genetics

## Abstract

Tumors may consist of billions of cells, which in malignant cases disseminate and form distant metastases. The large number of tumor cells formed by the high number of cell divisions during tumor progression creates a heterogeneous set of genetically diverse tumor cell clones. For cancer therapy this poses unique challenges, as distinct clones have to be targeted in different tissue locations. Recent research has led to the development of specific inhibitors of defined targets in cellular signaling cascades which promise more effective and more tumor-specific therapy approaches. Many of these molecular targeted therapy (MTT) compounds have already been translated into clinics or are currently being tested in clinical studies. However, the outgrowth of tumor cell clones resistant to such inhibitors is a drawback that affects specific inhibitors in a similar way as classical cytotoxic chemotherapeutics, because additionally acquired genetic alterations can enable tumor cells to circumvent the particular regulators of cellular signaling being targeted. Thus, it might be desirable to reduce genetic heterogeneity prior to molecular targeting, which could reduce the statistical chance of tumor relapse initiated by resistant clones. One way to achieve this is employing unspecific methods to remove as much tumor material as possible before MTT, e.g., by tumor debulking (TD). Currently, this is successfully applied in the clinical treatment of ovarian cancer. We believe that TD followed by treatment with a combination of molecular targeted drugs, optimally guided by biomarkers, might advance survival of patients suffering from various cancer types.

## Introduction

Cancer is a life-threatening disease and in western populations, about every third person is projected to suffer from cancer between birth and the age of 74 (1). Between different types of cancer and even among tumors of the same kind in different patients, strong variation can be observed in cell biology and genetics, so that the success of treatment approaches can be unpredictable and some patients fail to respond while others show full remission of the disease. Unraveling oncogenic cell signaling pathways and the underlying genetic alterations has led to the development of specific inhibitors of oncogenic signaling and the establishment of markers that indicate therapy success or failure. Despite initial success with molecular targeted drugs inhibiting oncoproteins or their downstream signaling molecules, resistance is frequently observed (2), as has been well-described for example in non-small-cell lung cancer (NSCLC) (3–5). Thus, advancing treatment regimens in a way to overcome drug resistance reliably is a substantial goal of cancer researchers and clinicians. Decades ago, it became apparent that single drugs or treatments against cancer are less efficient than combined approaches (6–8). Especially for many advanced tumor diseases, it will not be sufficient to use single agents in order to achieve substantial benefit for patients, as in the context of a large tumor burden the intratumoral heterogeneity recurrently creates resistant clones that can reestablish the disease after initial remission (9–11). It is much more promising to hit the neoplastic cells therapeutically from as many different sides as possible, so that the chance of combined resistance against all treatment approaches is reduced (12–14). Here, we discuss tumor debulking (TD) as a method to reduce clonal heterogeneity, which could synergize with the combined application of molecular targeted drugs.

## Tumor Size and Clonal Heterogeneity

Tumors in the human body vary strongly in their size, reaching from microscopic lesions to tens of kilograms of tissue. Tumor size has important implications for the genetic heterogeneity within the tumor, as an increase in the amount of neoplastic tissue requires cell divisions. Every new tumor cell, which acquires additional genetic hits and thus becomes genetically distinct from its parental cell, is seen as a new tumor cell clone (15). As tumor size and thus the contained number of cells expands, more and more genetically distinct clones are created. This happens in a statistical manner, as a given number of cell divisions at a given mutation rate has to result in a given average number of new mutations (16). However, this system is more complicated, as distinct tumor cell clones vary in their fitness and proliferation rate due to their distinct genetic conformation affecting tumor cell biology, and their location within the tumor resulting in a different access to oxygen and nutrients. This way, specific clones expand whereas others persist or become lost- a classical selection process of randomly created individuals by their natural characteristics known as “clonal evolution” [reviewed in (17)]. Moreover, cells that acquire new genetic alterations driving them to disseminate from the primary tumor and migrate to other tissue locations form distant metastases. Thus, in metastases, clonal evolution continues from the genetic conformation of the disseminated clone of the primary tumor and it has been possible for scientists to track how tumor cell clones have spread to different sites sequentially and which genetic changes were acquired along the metastatic path (18). This suggests that distant metastases tendentially have a higher mutational burden than the primary tumor.

New genetic alterations in subclones not only drive the metastatic spread, but also can confer drug resistance, e.g., by inhibiting cell death or activating cell signaling downstream of the targeted effectors (19, 20) [reviewed in (21, 22)]. Interestingly, especially small clones (under 10% of the tumor mass) frequently harbor treatment-resistant mutations and were observed more often in tumors with a slower growth rate (23). Thus, clonal heterogeneity within human tumors depends on a complex relation of tumor volume, growth rate, and time.

## Molecular Targeted Therapies for Precision Oncology

Standard treatment against most tumor diseases in clinics is mainly composed of surgery, radiotherapy and classical cytotoxic chemotherapy. Standard chemotherapeutic drugs inhibit cell division or damage DNA generally, which harms both normal and cancerous tissues. This causes side effects, mainly tissue defects, affecting quality of live and live-threatening secondary cancers (24). Large cohorts of new information on cancer genetics in the recent two decades have enabled the development of new tumor-specific approaches that inhibit key oncogenic signaling pathways. These so called “molecular targeted therapies^1^” (MTTs) include but are not limited to epidermal growth factor receptor (EGFR) monoclonal antibodies (e.g., cetuximab, panitumumab, zalutumumab, and nimotuzumab), EGFR tyrosine kinase (TK)-inhibitors (e.g., gefitinib, erlotinib, lapatinib, afatinib, and dacomitinib), vascular endothelial growth factor (VEGF) inhibitors (e.g., bevacizumab), or VEGF receptor (VEGFR) inhibitors (e.g., sorafenib, sunitinib, and vandetanib). Moreover, inhibitors of the PI3K/AKT/mTOR pathway, which is frequently aberrantly activated in various malignancies (e.g., rapamycin, temsirolimus, everolimus), have been developed recently (25). New cancer-targeting compounds also include specific inhibitors of the cell cycle [e.g., cyclin-dependent kinase (CDK)-inhibitors; reviewed in (26)] or inhibitors of epigenetic regulators of cell differentiation and proliferation [e.g., inhibitors of enzymes modifying DNA or histones by methylation/acetylation; reviewed in (27, 28)]. Moreover, PD-1/PD-L1 inhibitors in immunotherapy work as immune checkpoint inhibitors (ICIs) that target the tumor cells' ability to evade immune recognition and recently were described to be the preferable treatment option for a subset of patients with advanced or metastatic tumors (29).

As every individual tumor differs in its genetic alterations, therapies can be tailored to each patient's genetic alterations, which can cause a unique set of vulnerabilities against specific drugs/compounds. This strategy is called “precision oncology” [reviewed in (30)]. Recently, clinical oncologists have examined the relationship between the number of mutations detectable in a tumor disease and the number of mutations that indicate a vulnerability of the tumor toward a specific drug as a prognostic factor (31). This investigation has demonstrated that a patient‘s prognosis is more favorable, the more mutations carried by the individual tumor can be treated by specific drugs. This strikingly demonstrates the advantages of personalized treatment strategies over standard therapies based on general guidelines. However, the selection of molecular targeted drugs solely based on genetic alterations outside their established indications was shown to be ineffective in many cases and led to the conclusion that biomarkers for MTT efficiency have to be established further before individually matched MTT can become standard care (32–34). Thus, the presence of specific mutations verified as biomarkers in one tumor type might not work equivalently in another. For example, BRAF-inhibitors alone work efficiently in BRAF-V600E-positive melanomas as discussed below, but not in BRAF-V600E-positive colon cancer (35). Despite that overall conclusion, there are examples of genetic biomarkers that work universally. For instance, tropomyosin receptor kinase (TRK)-inhibitor larotrectinib inhibits all kinds of TRK-fusion-positive tumors in children and adults (36). As the DNA-sequencing technology further advances, it is highly probable that substantially more genetic markers will be described in the near future, so that precision oncology-based approaches might upgrade traditional treatment regimens in mid-term.

## Drug Specificity and Resistance

**Figure 1:**
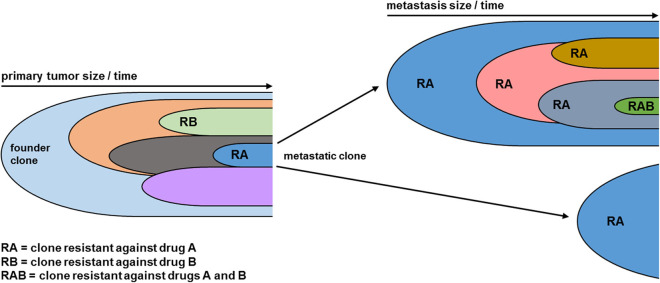
Clonal evolution leads to acquired combined drug resistance. As the tumor grows over time, new genetic alterations lead to the formation of distinct clones (indicated by distinct colors), each representing a subpopulation of the tumor mass. Clones vary in their topology within the tumor and their biology, ultimately leading to the formation of metastatic clones which initiate metastases at different locations. Moreover, new genetic hits lead to drug resistance against single drugs. As the tumor disease progresses, further individual clones can acquire a combined resistance against several drugs. As the genetic diversity statistically correlates with tumor volume, larger metastases **(Top Right)** are genetically more diverse than smaller metastases **(Left Right)**. Within metastases the clonal evolution continues from the metastatic clone of the primary tumor, leading to a higher mutational burden.

An important challenge for the clinical use of chemotherapeutics and new targeted drugs is the resistance of tumor cells. Tumor cells can be inherently resistant or acquire resistance. Even after a drug initially caused effective remission, single resistant tumor cell clones can survive and reestablish the tumor which then will be entirely resistant toward repeated treatment with the same drug. This phenomenon was observed recurrently [reviewed in (37)], especially when a single drug is used to treat a highly malignant type of cancer in an advanced stage. One reason for drug resistance is that above a certain tumor size, there will be clones that have the matching genetic alterations to resist the given drug (Figure 1). The important influence of resistant subclones on MTT has previously been discussed (38). How likely a cell can acquire resistance mainly depends on the drug‘s chemical structure, mode-of-action, and the target specificity (21, 39). It is obvious that a more specific drug which exclusively blocks one target protein, e.g., a single kinase, will be easier to resist than a compound with a broader impact, e.g., inhibiting all kinases, shutting down mitochondrial energy production, or blocking mRNA translation. Tumor cells can bypass a defined point in cellular signaling, e.g., by acquiring new mutations that activate downstream effectors. For this reason, resistance against single inhibitors used for MTT approaches can be observed frequently (4, 39). Even though it has been demonstrated that combined treatment with separate inhibitors can overcome tumor cell resistance (37, 40–42), a combined acquired resistance against several MTT drugs has been described as well (14).

## Tumor Debulking to Support Precision Oncology

**Figure 2:**
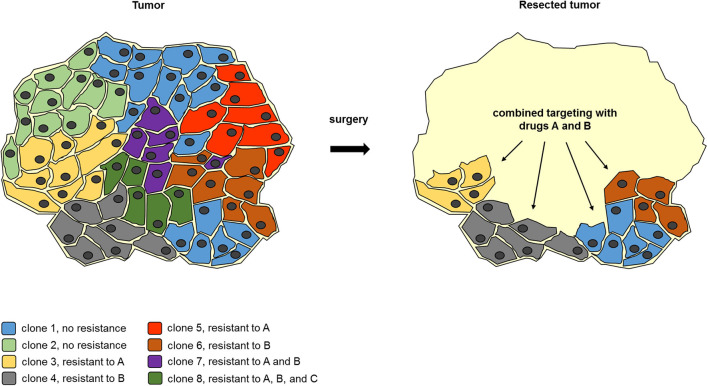
Example for surgery to reduce clonal heterogeneity synergizes with subsequent combined molecular targeting. A tumor body consists of a genetically heterogeneous set of tumor cell clones with a differential drug resistance spectrum. Upon partial removal of the tumor body, the number of tumor cell clones is reduced, so that a combined treatment with drugs A and B can eliminate the remaining tumor cells. This demonstrates how TD can facilitate subsequent molecular targeting even when residual tumor tissue remains.

As outlined above, clonal heterogeneity is positively linked to tumor burden and increases the chance of drug resistance. This indicates that reduction of tumor volume will elevate the likelihood of successful drug treatment. Clonal heterogeneity also plays an important role in acquired resistance against immunotherapy [reviewed in (43)]. However, as various cell types in the microenvironment participate in the complex interaction between tumor cells and the immune system, resistance against this kind of therapy is influenced by many more determinants [reviewed in (44)]. As TD surgery can reduce clonal heterogeneity by removing large tumor bodies, but cannot eliminate dispersed tumor cells, and MTT can target dispersed cells, but might find resistant clones in large tumor bodies, both treatments could work synergistically together when TD is performed prior to MTT (Figure 2).

Both treatments are already applied to treat ovarian cancer (OC) (45) and metastatic renal cell carcinoma (mRCC) (46). In OC targeted therapy, the VEGF-inhibitor bevacizumab or the VEGFR-inhibitors pazopanib (47) and cediranib (48) are used to inhibit angiogenesis. Similarly, PARP inhibitors are used to inhibit DNA-repair in tumor cells combined with chemotherapy which leads to the death of tumor cells (49, 50). Moreover, OC patients frequently undergo TD, as this has been shown to prolong the survival of OC patients if the residual nodules are <0.5 cm in size (51). For example, bevacizumab was shown to efficiently increase patient survival after TD surgery (45). In patients with mRCC, immunotherapy was shown to be much more effective in combination with TD than when applied alone (52).

Besides OC and mRCC, there is preclinical data indicating potential for the use of TD with MTT or chemotherapy in other tumor types as well. In a mouse model of malignant mesothelioma, TD was found to support anti-tumor memory when it was combined with chemotherapy and adjuvant immunotherapy (53, 54). In particular, one study demonstrated that a partial TD induced a long-term anti-tumor memory which was not observed when a complete resection was performed (53). Moreover, a partial tumor removal stimulated an anti-tumor immune reaction in a mouse model of NSCLC and it was shown that the excision of one tumor body increased the efficiency of anti-PD1 immunotherapy in another tumor location within the same individual (55). The reason for this might be the release of tumor antigens stimulating the immune system due to the destruction of tumor material along surgery and the decreased release of immunosuppressive cytokines from the reduced tumor tissue burden. This indicates that TD could be beneficial in combination with immunotherapy in different tumor types, even if a large proportion of the tumor burden remains. In addition to immunotherapy, TD was successfully combined with a cisplatin-loaded polymer platform in a mouse model of head and neck squamous cell carcinoma (56).

These examples indicate that TD could support therapy against a variety of cancer types. NSCLC, for example, is a cancer type with many molecular targeted drugs available in clinical practice. MTT for NSCLC includes drugs blocking receptor tyrosine kinases (RTKs) like EGFR, hepatocyte growth factor receptor (HGFR), and anaplastic lymphoma kinase (ALK), as these oncoproteins are frequently aberrantly activated. As described above, resistance against these new approaches in NSCLC therapy is frequently observed (3–5) and thus new strategies to overcome resistance are needed. Surgery is regularly used for locoregionally advanced lung cancers, but usually not combined with MTT in clinical routine even if clear biomarkers for targeted drugs are detected. Beneficial results in applying MTT after NSCLC surgery have been observed in initial clinical trials [reviewed in (57)]. In a single-arm phase II trial, the EGFR-inhibitor erlotinib was applied in patients with stage IA-IIIA NSCLC with EGFR-mutation after surgery, which resulted in an increased 2-year survival and a block of recurrence during the period of drug application, so that recurrence was delayed in most cases until treatment was discontinued (58). However, this indicates that erlotinib was not sufficient to kill all remaining tumor cells, but inhibited the surviving fraction to repopulate the tumor while being administered. Using an even more effective MTT, e.g., a combination of several targeted drugs, TD could support MTT against NSCLCs, maybe even in advanced stages, and minimize the chance of relapse by reducing the number of potentially resistant tumor cells. This could be further tested in clinical trials with combinations of targeted drugs, dividing patients with NSCLC who have an indication for MTT into two groups. One group would receive MTT in the conventional way, whereas the patients in the second group undergo TD prior to drug administration. By setting the proportion of residual tumor material after debulking in correlation to the therapy success, this study could reveal how reasonable an incomplete tumor resection is for this tumor type.

## Personalized Application of Tumor Debulking

As TD represents an unspecific mechanical method, the reduction of genetically distinct tumor cell clones by TD could significantly support all therapy approaches, which are limited in their efficacy by tumor cell resistance mediated by genetic variation within the tumor. If this is true, TD will support MTT regardless of the specific drug's mode-of-action by lowering the chance of resistance. Newer MTT approaches consider the genetics of individual tumor diseases and match molecular targeted drugs to the genetic profile of the tumor (31). This indicates that if it was possible to measure the effect that TD has on genetic heterogeneity, e.g., using a liquid biopsy or similar procedures, the MTT design could be created after TD to benefit maximally from the improved tumor genetics. Liquid biopsies are designed to detect circulating tumors cells, circulating tumor DNA (ctDNA), and other tumor-derived components in a patient's blood sample (59). In a clinical study ctDNA detection rates of >75% were observed for many cancer types, with ~50–75% even in cases with localized disease (60). Interestingly, the authors detected driver mutations in the *KRAS* oncogene in ctDNA as well as mutations related to the development of resistance toward EGFR blockade in 23 of 24 patients that initially responded but later relapsed. This indicates that liquid biopsy is a sensitive method for analyzing the tumor genome and tailoring MTT to each patient individually. Liquid biopsy might even enable a comparison of the tumor genome before and after TD, so that the impact of TD on clonal heterogeneity could be monitored.

Of course, the application of TD in order to decrease clonal heterogeneity would make sense especially if promising MTT options can be identified for the particular patient. An example of a highly potent MTT is BRAF-inhibition in melanoma which initially works highly effectively and can eradicate even large tumors, but in most cases induces resistance due to alternative activation of MAPK/Erk signaling or activation of PI3K/Akt signaling [reviewed in (61)]. Even combined inhibition of BRAF and MEK was followed by relapse, despite a significantly longer survival compared to single BRAF-inhibitor treatment (62). This indicated that effective treatment, even in combination, most frequently faces resistant tumor cell clones in advanced diseases. Thus, TD prior to BRAF/MEK-inhibitor application might be effective in melanoma treatment. This hypothesis is supported by a clinical phase III trial that reported a significantly decreased recurrence of completely resected, stage III melanoma with *BRAF*-V600E or -V600K mutations treated with a combination of BRAF and MEK inhibitors after surgery (63).

Due to higher tumor volumes and the resulting higher genetic heterogeneity, advanced stage tumor patients might benefit more likely from TD (29). However, surgery-related mortality and morbidity have to be considered to estimate for every patient individually whether the expected benefits of the planned MTT are high enough to justify the operation risks and negative impact on life quality. In the scenario when TD is not possible to perform due to excessive risks, MTT might be combined with other treatments like chemotherapy, radiotherapy, hyperthermia, or others to achieve a cytoreductive effect that will reduce the chance of resistance against MTT. However, in our view, TD is not primarily supposed to change how MTT is performed, but rather support it whenever possible. Hence, TD to support MTT must be performed as intensely as reasonably safe.

## Conclusions

Preclinical and clinical studies indicate that TD might cooperate well with MTT approaches. Immunotherapy approaches in particular have been shown to benefit from tumor resection in a large variety of tumor types. The reduction of as many genetically distinct tumor cell clones as possible could be used to reduce the ability of tumors to resist MTT for precision oncology. In order to create synergy effects, unspecific non-mutagenic treatment options like TD should precede genetics-guided combined molecular targeting for a variety of tumor types. Depending on the individual patient's characteristics, tumor type, stage, and genetic profile, oncologists could design a personalized strategy to support specific treatment options like MTT with cytoreductive methods like TD to outsmart the tumor's intrinsic compulsion to resistance. In future, clinical treatment guidelines might be adapted this way to facilitate an effective patient-specific MTT.

## Author Contributions

FO developed the idea and created the design of the article. HS and MG contributed to the idea. All authors participated in writing and editing the manuscript.

## Conflict of Interest

The authors declare that the research was conducted in the absence of any commercial or financial relationships that could be construed as a potential conflict of interest.

## Footnotes

^1^https://www.cancer.gov/about-cancer/treatment/types/targeted-therapies/targeted-therapies-fact-sheet
